# Chromosome Translocation t(6; 14) With Different Phenotypes and Segregation Patterns: A Report of Two Cases

**DOI:** 10.7759/cureus.68402

**Published:** 2024-09-01

**Authors:** Ravindran Ankathil, Wan Nur Amalina Zakaria, Mohd Ridzuan Hamid, Siti Mariam Ismail, Nazihah Mohd Yunus, Aziati Azwari Annuar, Hans Van Rostenberghe

**Affiliations:** 1 Department of Cytogenetics and Genomics, Jubilee Centre for Medical Research, Jubilee Mission Medical College and Research Institute, Thrissur, IND; 2 Central Research Laboratory, PMS College of Dental Sciences and Research, Trivandrum, IND; 3 Human Genome Centre, School of Medical Sciences, Universiti Sains Malaysia Health Campus, Kota Bharu, MYS; 4 Department of Pediatrics, School of Medical Sciences, Universiti Sains Malaysia Health Campus, Kota Bharu, MYS

**Keywords:** translocations 6 & 14, abnormal chromosomes, rare translocation, tertiary trisomy, congenital abnormalities

## Abstract

Chromosomal rearrangement can disrupt gene function by interfering with coding sequences or their regulatory regions. The breakpoint in these rearrangements can pinpoint the disease-related gene's location. This paper presents two rare cases of chromosomal rearrangement involving chromosome 6 (6p24-25) and chromosome 14 (14q22-23). The first case involves a girl with hearing impairment, inheriting a balanced translocation of chromosomes 6 and 14 from her father. The second case describes a dysmorphic baby boy with congenital bilateral choanal atresia and a tertiary trisomy, involving a translocation between chromosome 6 (6p24) and chromosome 14 (14q22), resulting in a derivative chromosome (14) in addition to the normal complement of chromosomes 6 and 14. The boy’s mother had a history of four recurrent miscarriages. However, the origin of this tertiary trisomy in the second case presented could not be delineated because the parents did not consent and declined their blood samples for karyotyping. Parental karyotyping and chromosomal analysis are crucial for investigating recurrent miscarriages, identifying genetic causes, guiding reproductive decisions, and improving successful pregnancy outcomes for affected couples.

## Introduction

Structural abnormalities involving translocations and inversions of human chromosomes occur in around 0.5% of newborn infants [1]. These chromosome rearrangements can either be balanced or unbalanced. Among the balanced chromosomal rearrangements (BCR), the estimated frequency of reciprocal translocations was 1/560, whereas that of inversions was 1/1,100 [2].

In BCR, the chromosome complements remain complete, with no loss or gain of genetic material. Therefore, they are typically harmless, except in rare cases where a breakpoint disrupts an important functional gene. Problems can arise during meiosis when chromosomes involved in the translocation cannot pair normally to form bivalents. Instead, they form a pachytene quadrivalent, where each chromosome aligns with homologous material. During meiotic divisions, they can undergo either a 2:2, 3:1, or 4:0 segregation pattern [3].

When chromosomes in the quadrivalent separate during the later stages of meiosis I, they segregate differently. If alternate segregation occurs (when alternate chromosomes segregate to each gamete), the gamete will carry a normal or balanced haploid complement. Upon fertilization, the embryo will either have normal chromosomes or carry a balanced rearrangement [4]. In contrast, adjacent segregation (where adjacent chromosomes segregate together) results in an unbalanced chromosome complement [3]. Unbalanced chromosomal rearrangements can lead to syndromes, multiple congenital anomalies, and/or intellectual disabilities [5].

Although the majority of BCR carriers do not exhibit abnormal phenotypes, 6% of balanced translocations and 9.4% of balanced inversions are associated with abnormal phenotypes [6]. We report two rare cases of chromosomal rearrangement involving the short arm of chromosome 6 (6p24-25) and the proximal segment of the long arm of chromosome 14 (14q22-23).

## Case presentation

Case 1

A nine-year-old Chinese girl, the second of three siblings born to parents aged 42 and 39 years, was referred for bilateral sensorineural hearing loss and clitoromegaly. She was born at term with normal birth weight and no congenital anomalies. The family history was unremarkable, with no instances of deafness or intellectual disability among her parents and siblings.

**Figure 1:**
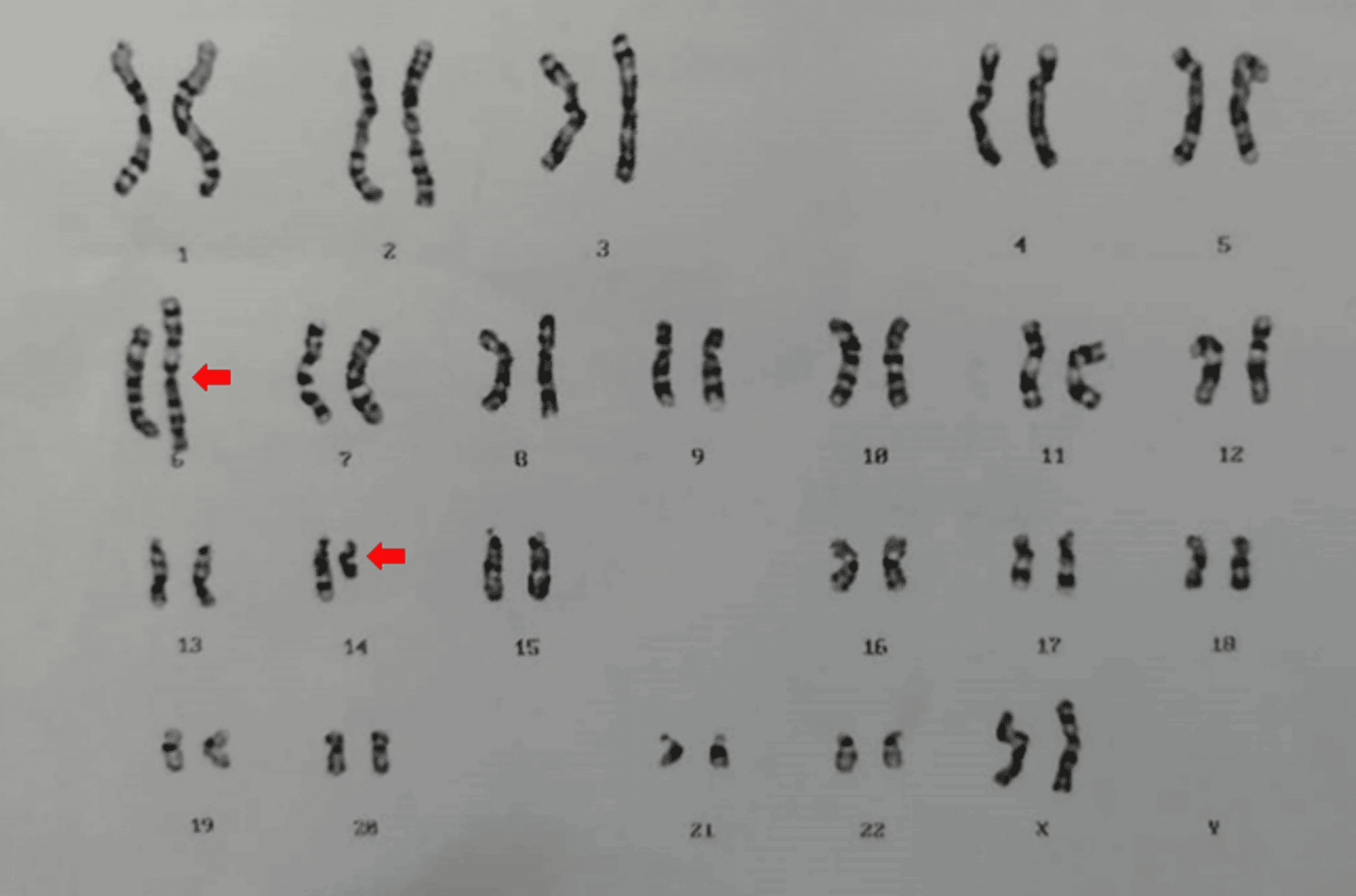
The karyogram of Case 1 identified by the G-banding technique showing the chromosome constitution 46,XX,der(6)t(6;14)(p25;q23) karyotype pattern. A derivative chromosome 6 has resulted from a balanced translocation of the chromosome 14 segment distal to 14q23 to the short arm of chromosome 6 at band 6p25. The arrow indicates the abnormal chromosome.

Cytogenetic analysis of a peripheral blood sample, conducted at the Genetic Laboratory of the Human Genome Centre, Hospital Universiti Sains Malaysia, revealed an abnormal 46,XX,der(6)t(6;14)(p25;q23) karyotype (Figure 1). This indicated a derivative chromosome 6 resulting from a balanced translocation, where the distal segment of chromosome 14 at 14q23 was translocated to the short arm of chromosome 6 at 6p25.

**Figure 2:**
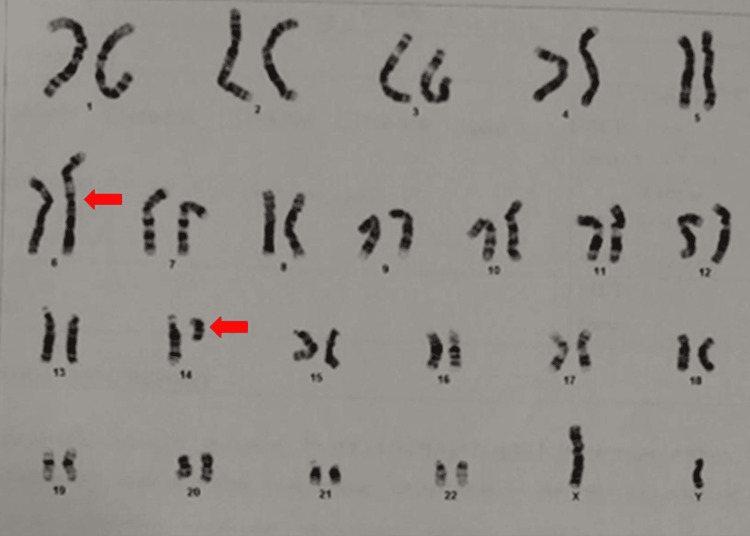
Parental chromosome studies of Case 1 revealed an abnormal karyotype 46,XY, der(6)t(6;14)(p25;q23) confirming the direct inheritance of der(6)t(6;14)(p25;q23) from the father. The arrow indicates the abnormal chromosome.

Parental karyotyping showed the father had an abnormal karyotype 46,XY,der(6)t(6;14)(p25;q23), confirming the direct inheritance of the derivative chromosome from the father (Figure 2). The mother's karyotype revealed the 46,XX normal female karyotype pattern.

Case 2

A 9-day-old Chinese boy, the second viable child out of six pregnancies from a non-consanguineous marriage, was born at 38 weeks gestation via elective lower segment cesarean section with a low birth weight of 2.3 kg. The first viable offspring was a healthy three-year-old girl. Both parents are 37 years old with a history of four recurrent miscarriages.

At birth, the baby was not vigorous, cyanosed, and had poor Apgar scores, requiring intubation. During intubation, the attending doctor noted the inability to pass the scope through bilateral nostrils, likely due to bilateral choanal atresia. Other dysmorphic features included a wide anterior fontanelle, hypertelorism, flat nasal bridge, low set and posteriorly rotated ears, small face, small chin, left structural congenital talipes equinovarus (CTEV), and symmetrical small size for gestational age (SGA).

**Figure 3:**
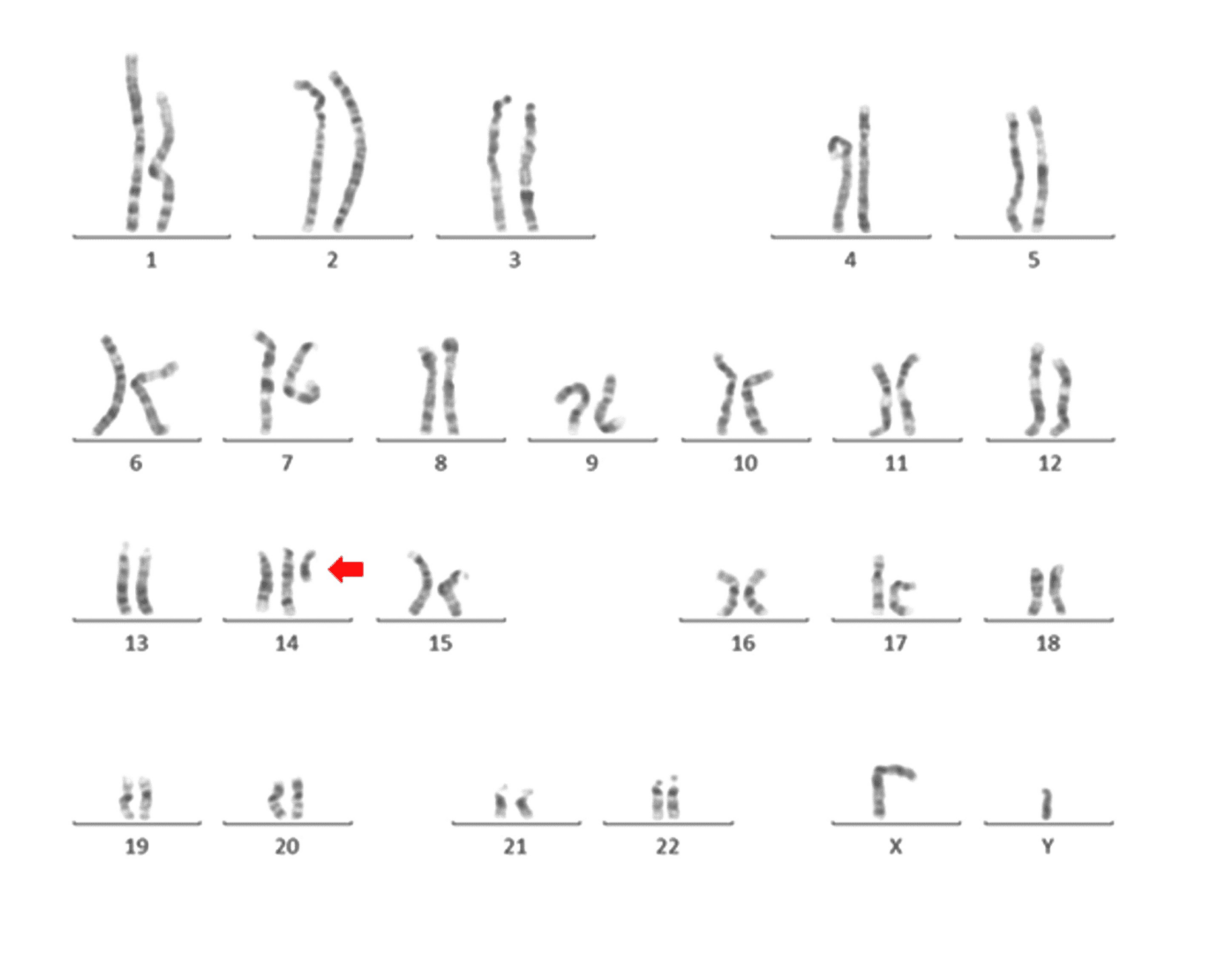
The karyogram of Case 2 identified by the G-banding technique showing the chromosome constitution 47,XY,+der(14)t(6;14)(p24;q22). The arrow indicates the abnormal chromosome.

The chromosome analysis of the Case 2 patient revealed an abnormal 47,XY,+der(14)t(6;14)(p24;q22) karyotype pattern as shown in Figure 3. An abnormal male karyotype with 47 chromosomes, consisting of an extra small marker chromosome, was identified as a derivative chromosome 14 on further analysis. The extra derivative chromosome 14 is made up of 6p24 and 14q22 probably resulted from a translocation of chromosome 6 segment 6p24 to the segment 14q22 of chromosome 14. The karyotype contains two normal copies of chromosomes 6 and 14 and extra copies of segments of chromosome 6p24 till 6p25.3 and chromosome 14q10 till 14q22 resulting in a numerically unbalanced partial trisomy karyotype. 

**Figure 4:**
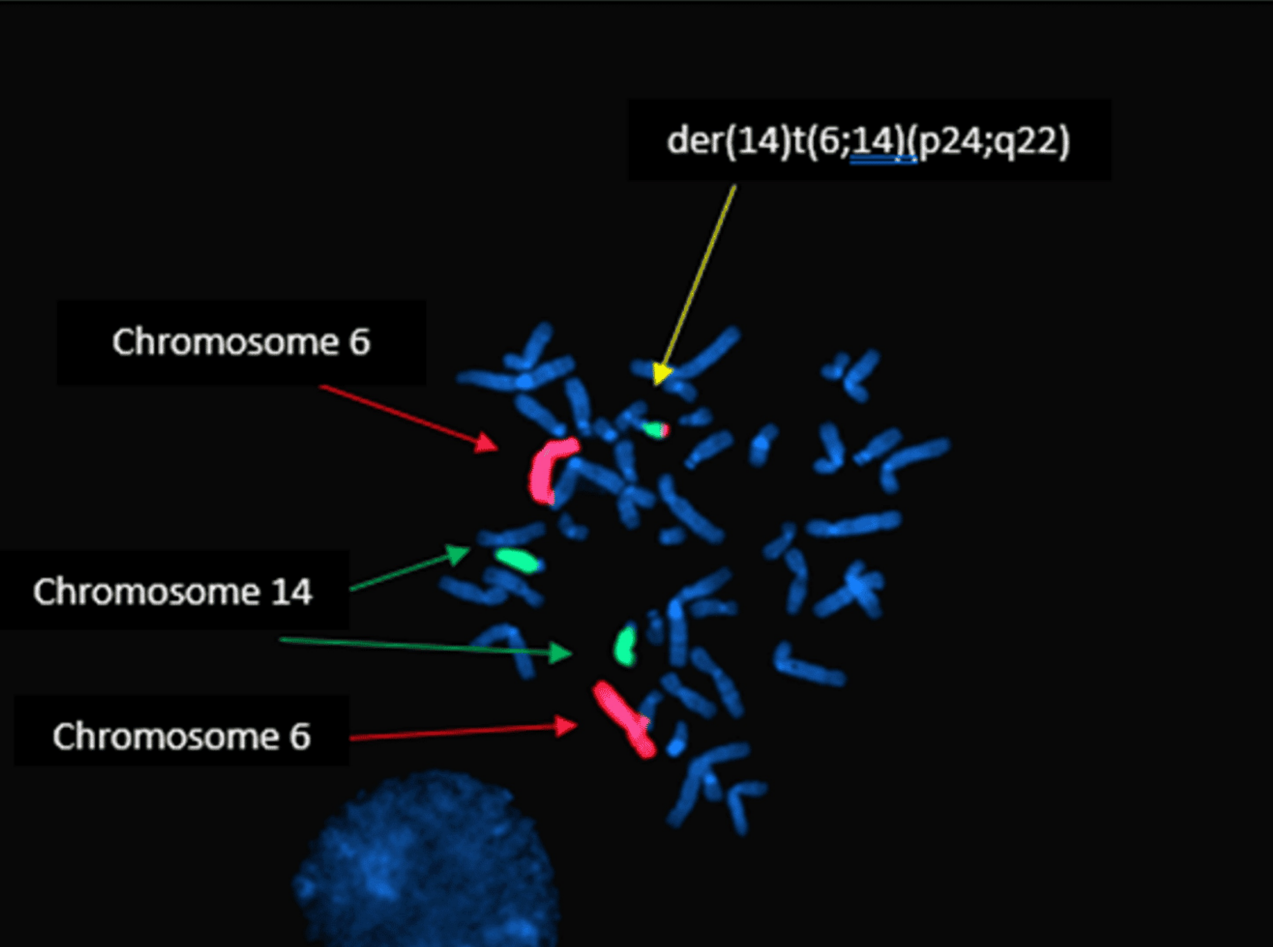
The origin of the extra chromosome in Case 2 was further evaluated by molecular cytogenetic analysis employing the FISH technique using a WCP for chromosome 6 (red signals) and 14 (green signals) confirming the extra chromosome derived from segments of chromosome 6 and 14 FISH: fluorescence in-situ hybridization; WCP: whole chromosome painting probe

As the parents of Case 2 declined their samples to be karyotyped, it was not possible to delineate whether the abnormal karyotype was inherited or de novo in origin. Fluorescence in-situ hybridization (FISH) analysis using a whole chromosome painting probe (WCP) for chromosomes 6 and 14 confirmed that the extra derivative chromosome was derived from segments of chromosomes 6 and 14 (Figure 4).

## Discussion

The karyotype of Case 1 was 46,XX,der(6)t(6;14)(p25;q23). Balanced translocation on different segments of chromosomes 6 and 14 has been reported in a patient with intellectual disability and agenesis of the corpus callosum with karyotype 46,XY,t(6;14)(q26;q13)dn by Back et al. (2011) [7]. The case reported by Back et al. (2011) was de novo in origin, whereas our reported Case 1 was found to be inherited from the father. It is reasonable to postulate that the translocation breakpoints of the chromosome involved might have disrupted the coding of an important functional gene or resulted in a positional effect affecting other genes and might have contributed to Case 1’s phenotype, especially hearing impairment. The segment distal to 14q23 of chromosome 14 has been translocated to the short arm of chromosome 6 at band 6p25.

The UCSC (University of California, Santa Cruz) genome browser and Online Mendelian Inheritance in Man (OMIM) database indicate that the 6pterp25.1 region contains 26 coding genes. Notably, mutations in FOXC1 can lead to an autosomal dominant Axenfeld-Rieger anomaly with cardiac defects and/or sensorineural hearing loss. Additionally, mutations in SERPINB6 and RIPK1 are associated with autosomal recessive (ar) deafness (#613453) and immunodeficiency (#618108), respectively [8]. The DFNB35 (#608565) gene, related to autosomal recessive hearing impairment, is located on chromosome 14 at 14q24 and is involved in the reported translocation of Case 1. It is postulated that the translocation breakpoints of the chromosome involved might have disrupted the coding of this gene, resulted in a positional effect affecting other genes, and contributed to this patient's phenotype, especially hearing impairment. Further molecular studies are needed to explore how these genes contribute to hearing impairment due to the observed translocation.

The karyotype of Case 2 was 47,XY,+der(14)t(6;14)(p24;q22). This abnormal male karyotype includes an extra-small marker chromosome, identified as a derivative chromosome 14. This derivative chromosome 14 comprises segments 6p24 and 14q22, likely resulting from a translocation of chromosome 6 segment 6p24 to chromosome 14 segment 14q22. The karyotype also contains two normal copies of chromosome 6p24p25.3 and chromosome 14q10q22, resulting in a numerically unbalanced partial trisomy, or tertiary trisomy. Although parental karyotyping was not performed due to parental reluctance, it is reasonable to infer that one parent may be a balanced carrier of the t(6;14) translocation, given the mother's obstetric history.

Partial trisomy of the short arm of chromosome 6 is rare and associated with a variable phenotype. Therkelsen et al. first described such cases involving unbalanced translocations between chromosomes 6p and 20p [9]. Villa et al. provided a comprehensive review of similar cases [10]. The proximal breakpoint on the short arm of chromosome 6 varies from 6p11 to p25. Clinical features of duplications include low birth weight, developmental delay, craniofacial abnormalities, feeding difficulties, and congenital defects. Characteristic craniofacial findings include craniosynostosis, a prominent forehead, and choanal atresia. Intellectual disability and other developmental issues are also reported [11].

The short arm of chromosome 6 contains approximately 1900 genes, with about 140 in region 6p23p25.3. Genes such as BMP6, EDN1, and F13A1 are potential candidates for contributing to the observed phenotypes in cases of 6p distal trisomy. BMP6, for instance, is involved in early embryonic development and may contribute to craniofacial abnormalities [12]. 

Kovacs and Mihai described a case of tertiary trisomy 14q due to a paternal balanced translocation [13]. Patients with trisomy 14pter~q22 show growth retardation, craniofacial dysmorphism, and other anomalies. Variations in phenotype may be attributed to additional trisomic or monosomic chromosome segments [14].

## Conclusions

In conclusion, these pioneering case series highlight two exceptionally rare instances of chromosomal malsegregation involving breakpoints on the short arms of chromosomes 6 and 14. Despite the similarities in the affected regions, the cases exhibit distinct segregation patterns. These case series underscore the significant variability in phenotypic outcomes associated with these chromosomal anomalies and provide crucial insights into their rare but impactful nature.
